# Supplementary material for: Prevalence and genetic variability of occult hepatitis B virus in a human immunodeficiency virus positive patient cohort in Gondar, Ethiopia
Source: PLoS One. 2020 Nov 19;15(11):e0242577. doi: 10.1371/journal.pone.0242577 (PMC7704059; doi:10.1371/journal.pone.0242577)
Supplement: Attachment — Submitted filename: Response to the reveiwers_Oct, 2020_.docx [file pone.0242577.s001.docx]

October 5, 2020

Professor Isabelle Chemin

Editor, PLOS-ONE

Dear Dr. Chemin

We would like to thank the Editor and the reviewers for their feedback on our manuscript.

We have enclosed a point-by-point response to address the reviewer comments. All authors have seen and approved the final version of the manuscript.

Sincerely,

Tekalign Deressa, PhD

Ethiopian Public Health Institute

**Authors’ Response to the Reviewers Report**

1. Please ensure that your manuscript meets PLOS ONE's style requirements, including those for file naming. The PLOS ONE style templates can be found at

<https://journals.plos.org/plosone/s/file?id=wjVg/PLOSOne_formatting_sample_main_body.pdf> and

<https://journals.plos.org/plosone/s/file?id=ba62/PLOSOne_formatting_sample_title_authors_affiliations.pdf>

**Thank you. We have strictly adhered to the journal’s style requirement.**

2. Please provide additional details regarding participant consent.

In the ethics statement in the Methods and online submission information, please ensure that you have specified (i) whether consent was informed and (ii) what type you obtained (for instance, written or verbal, and if verbal, how it was documented and witnessed).

If the need for consent was waived by the ethics committee, please include this information.

**In this version, the issue regarding patient consent is clearly described in the method section please check. FYI, we obtained a written informed consent.**

3. Your ethics statement must appear in the Methods section of your manuscript. If your ethics statement is written in any section besides the Methods, please move it to the Methods section and delete it from any other section. Please also ensure that your ethics statement is included in your manuscript, as the ethics section of your online submission will not be published alongside your manuscript.

[Note: HTML markup is below. Please do not edit.]

**Thank you! Done**


Reviewers' comments:

Reviewer's Responses to Questions

**Comments to the Author**

1. Is the manuscript technically sound, and do the data support the conclusions?

The manuscript must describe a technically sound piece of scientific research with data that supports the conclusions. Experiments must have been conducted rigorously, with appropriate controls, replication, and sample sizes. The conclusions must be drawn appropriately based on the data presented.

Reviewer #1: Yes

2. Has the statistical analysis been performed appropriately and rigorously?

Reviewer #1: Yes

3. Have the authors made all data underlying the findings in their manuscript fully available?

The [PLOS Data policy](http://www.plosone.org/static/policies.action#sharing) requires authors to make all data underlying the findings described in their manuscript fully available without restriction, with rare exception (please refer to the Data Availability Statement in the manuscript PDF file). The data should be provided as part of the manuscript or its supporting information, or deposited to a public repository. For example, in addition to summary statistics, the data points behind means, medians and variance measures should be available. If there are restrictions on publicly sharing data—e.g. participant privacy or use of data from a third party—those must be specified.

Reviewer #1: Yes

4. Is the manuscript presented in an intelligible fashion and written in standard English?

PLOS ONE does not copyedit accepted manuscripts, so the language in submitted articles must be clear, correct, and unambiguous. Any typographical or grammatical errors should be corrected at revision, so please note any specific errors here.

Reviewer #1: Yes

5. Review Comments to the Author

Please use the space provided to explain your answers to the questions above. You may also include additional comments for the author, including concerns about dual publication, research ethics, or publication ethics. (Please upload your review as an attachment if it exceeds 20,000 characters)

Reviewer #1: The authors described the prevalence of occult hepatitis B infection (OBI) in HIV infected patients, as well as their genotypes and mutations in S, P and pre-C-C regions. The study was conducted on 308 HIV + patients’ in Gondar, Ethiopia. The authors had already assessed the prevalence of HBsAg in this same cohort in 2017. OBI was identified in 291 HBsAg negatives HIV infected patients. Obove them 115 (39.5%) were anti-HBc (+) with an OBI prevalence over 19% (22/115 patients).
Male gender groups seem to be the most affected (prevalence not calculated by the authors) and are all of D genotypes.
Genotype D has been reported to be predominant. Mutations associated with HBV reactivation were detected in 3/22 OBI patients. The most common resistant mutation rtM204V / I (lamivudine), affecting the C domain of the polymerase by transforming the YMDD motif into YVDD or YIDD was found in one treatment-naive patient. The authors show the importance of diagnosing OBI in HIV infected patients.

**We are very thankful to the reviewers for the constructive comments and suggestions. In the section below, we provide a point-by-point response to each comments/suggestions.

Material and methods**
Line 106-107: Patient recruitment period was not specified.

**Thank you for the comment. The study subjects were recruited from March 2016 to July 2016.**

Lines 174-175; 196-197: legends of Figures 1 and 2 are not at their appropriate place. They should be placed under the corresponding figures.

**Thank you. The legends for Figures 1 and 2 now appear directly after the paragraphs in which they are first cited.**

Primers sequences and amplified fragments size were not specified in the methodology.

**Thank you. The primer sequences and size of amplified fragments are now specified in the methods sections (lines 128-133).**

Statistical analyzes:
the p value should be specified.

**Thank you. The p value is specified, please see over.**

Results:
Line 146-154 and table 1: We understand that it is the same cohort, but the same results have already been published by the same team in 2017 as well as all the results of table 1, which already appeared in table 1 of the ref 15. A summary of these results might be sufficient.
The authors should focus on new findings specific to the sub-cohort where OBI is reported.
**Thank you. This comment has taken into consideration. We have made some adjustment to avoid unnecessary duplication. However, we feel that some background information on demographics and laboratory data would help the readers to understand the subject without referring to other sources and included in this version too. As well, lifestyle risk data in this table was not included in our 2017 publication.**

Image quality of phylogenetic tree could be improved. It will help to differentiate the patients’ sequences from references. Highlighting the patients’ sequence when editing the phylogenetic tree would improve clarity.

**Thank you. We have uploaded higher quality images of the phylogenetic trees and figure 1 onto the PACE digital diagnostic tool. Both figures 1 and 2 images now meet the PLOS requirements.**

- It would be interesting to see viral loads results.

**Unfortunately, we do not have a viral load result and we could not include it as we had left with an insufficient volume of each specimen.**

- Was co-infection with hepatitis C investigated?

**Thank you. Co-infection with hepatitis C was not investigated. Testing for hepatitis C in HIV positive patients is not standard of care in Gondar, Ethiopia. As well, we want to focus more on hepatitis B as our main aim is to determine the prevalence and genetic variability of occult hepatitis B in HIV positive patients.**

- Did the authors find deletions in S region?

**Thank you. Deletions in the S region were not found.**

Ligne 223: Cote d’ Ivoire (french) = Ivory Coast

**Corrected!**

References
Reference 2: authors list doesn’t appear on the reference 2.

**Thank you! Corrected.**

6. PLOS authors have the option to publish the peer review history of their article ([what does this mean?](https://journals.plos.org/plosone/s/editorial-and-peer-review-process#loc-peer-review-history)). If published, this will include your full peer review and any attached files.


If you choose “no”, your identity will remain anonymous but your review may still be made public.


**Do you want your identity to be public for this peer review?** For information about this choice, including consent withdrawal, please see our [Privacy Policy](https://www.plos.org/privacy-policy).

Reviewer #1: No
